# Intestinal parasitic infections and associated factors among mentally disabled and non-disabled primary school students, Bahir Dar, Amhara regional state, Ethiopia, 2018: a comparative cross-sectional study

**DOI:** 10.1186/s12879-019-4165-2

**Published:** 2019-06-21

**Authors:** Agumas Ayalew Fentahun, Anemaw Asrat, Abebayehu Bitew, Selamawit Mulat

**Affiliations:** 1 Family Guidance Association of Ethiopia, Bahir Dar Model Sexual and Reproductive Health Clinic, Bahir Dar, Ethiopia; 2 School of Public Health, College of Medicine and Health Sciences, Bahir Dar University, Bahir Dar, Ethiopia; 3 University of Gondar, and Health, Injibara, Ethiopia; 4 Injibara Woreda Health Office, Injibara, Ethiopia

**Keywords:** Mental disability, Intestinal parasitic infection, Primary school students

## Abstract

**Background:**

Intestinal parasitic infections are still common in low-income countries including Ethiopia, particularly in children due to low-quality drinking water, poor personal and environmental sanitation. Disabled individuals are excluded from most academic, economic, social and cultural opportunities, they are among the poorest and most marginalized of the whole world’s people.

The aim of this study was to assess the prevalence of intestinal parasitic infections and associated factors among mentally disabled and non-disabled students at primary schools in Bahir Dar city, Amhara regional state, Ethiopia, 2018.

**Methods:**

A school-based Comparative cross-sectional study design was conducted from November 1–30, 2018. A total of 418 study participants, 104 mentally disabled and 314 non-disabled students were recruited through a simple random sampling technique. The collected data were coded, entered and cleaned with EpiData version 3.1 and analyzed using SPSS version 23. Multivariable logistic regression was conducted to identify factors associated with intestinal parasitic infections. The adjusted odds ratio with a 95% Confidence interval at a 5% level of significance was used to measure the strength of association.

**Results:**

The mean age of study participants was 14.05 ± 3.66 and 11.96 ± 2.94 for mentally disabled students and non-disabled students. Prevalence of parasitic infection was 56.70% (*n* = 59) for mentally disabled students whereas 41.10%(*n* = 129) for non-disabled students. Unclean fingernails [AOR = 2.42; 1.40,4.17], health checkups [AOR = 1.87;1.16,3.02], hand washing with water only [AOR = 2.48; 1.49,4.12], cooking and sanitation source of water [AOR = 4.40;2.32,8.36], Grade [ (1–4)] [AOR = 2.27;1.41,3.67], sex [AOR = 1.64;1.03,2.63] and Family size> = 7 [AOR = 2.74;1.25,5.99] were variables which showed statistically significant association with intestinal parasitic infections.

**Conclusion:**

The prevalence of intestinal parasitic infection was higher among mentally disabled students than non-disabled students. Unclean fingernails, health checkups, hand washing habits, source of water, family size, sex and Grade of students have had a statistically significant association with intestinal parasitic infections. Periodic medicinal treatment was needed twice a year for mentally disabled and once a year for non-disabled students.

**Electronic supplementary material:**

The online version of this article (10.1186/s12879-019-4165-2) contains supplementary material, which is available to authorized users.

## Background

Parasites are defined as organisms that get food and shelter from other organisms or the host and often harm it. For a parasitic to be outlined as intestinal, it must have an intestinal life cycle stage. Additionally, it may have a life cycle stage in the heart, circulation, lung, tissue, and other animals on the surrounding [1]. The most routes of entry of intestinal parasites into the human body are ingestion, skin penetration, aspiration, and auto-infection. Ingestion (fecal-oral routes); contamination of water and food is generally the foremost style of transmitting protozoan infections [2].

Intestinal parasitic infections (IPIs) comprise both helminths and protozoan which form the most common infections worldwide [3]. Helminths are worms with many cells while protozoa are parasites which have only one single cell and can multiply inside the human body [4] Most frequent Intestinal helminths were *A. lumbricoides*, *E. vermicularis*, *Hymenolepis species*, *T. trichiura*, *E. vermicularis*, *S. stercoralis*, *Hookworm*, *S. mansoni,* and *Tania species*, while most frequent intestinal protozoa include *E. histolytic* and *G. intestinalis* [5].

Globally, IPIs remains endemic. About one-third of the world’s population, more than 2 billion people are infected by IPIs, with the most being children [6, 7]. Similarly, in South Africa, primary school children of Eastern Cape Province 64.8% study participants were positive for ova and cysts of parasites from which 57.4% were known pathogenic parasites [8]. Among students in Kigali, Rwanda More than half 50.5% of the stools examined were infected with an intestinal parasite [9]. In Ethiopia too, there were different studies conducted in different regions at different time periods to assess the PIPIs; of which in Gamo area prevalence of intestinal parasitic infections was 39.9% [10] while in Chencha town 81.0% [11].

Disability is the umbrella term for impairments, activity limitations and participation restrictions, pertaining to the negative aspects of the interaction between a personal (with a health condition) and that individual’s contextual factors [12]. They were routinely excluded from most academic, economic, social and cultural opportunities, they’re among the poorest and most marginalized of the world’s young people [13]. Due to the above factors, improper sanitary hygiene and illiteracy about personal hygiene People with disabilities have a higher risk of IPIs than non-disabled individuals [14].

Although they have a higher risk of IPIs due to self and family care problems, there were no published studies on the burden of IPIs among people with disabilities in Ethiopia. Therefore, the aim of this study was to assess the prevalence of Intestinal parasitic infections and associated factors among mentally disabled and non-disabled students in Bahir Dar city at primary schools’ students.

## Methods

### Study area

The study was conducted at Bahir Dar city among students at public primary schools which contain mentally disabled and non-disabled students inclusively. Bahir Dar was one of the fast-growing cities in the country and serves as capital city of Amhara regional state which located 563 km away from the North West of Addis Ababa, the capital city of Ethiopia, having a total Area of 28 km^2^, latitude and longitude of 11°36′N 37°23′E and an elevation of 1840 m (6,040 ft. feet) above sea level. The city had four hospitals (2 private), ten health centers and different private profitable and non-profitable health institutions. Primary schools in Bahir Dar city have not sufficient latrine lavatories and poor in environmental sanitation. In Bahir Dar, mentally disabled students registered well and attained their education as special needs at primary schools however still they are at first cycle primary schools due to their mental disability problem. So, Bahir Dar city was the real setting to conduct this comparative study among mentally disabled and non-disabled primary school students. According to Bahir Dar city Educational office, there were 122 primary school students registered as mentally disabled and 6816 non-disabled primary school students attending in those four primary schools [15]. A school-based comparative cross-sectional study design was conducted from November 1 to November 30, 2018.

### Study population

Mentally disabled students: students registered as had being an intellectual problem at primary schools during the study period.

Non-disabled students: Students who had not any form of disability and attending primary schools during the study period.

### Sample size determination and sampling technique

The required final sample size was calculated using double population proportion formula: Two groups have unequal sample size (1:3, Disabled to nondisabled ratio), with the assumptions of 95% confidence level, power of 80, 5% of marginal error, 10% non-response rate, and prevalence of IPIs among non-disabled students (P2 = 65.5%), from the previous study conducted in Bahir Dar city, Dona Berber primary school students [16] and the prevalence of IPIs among mentally retarded students in the previous study is not known; Therefore we took P1 = 50%as prevalence for disabled students based on the assumption of getting greater sample size with *p* = 50%. Therefore, the final required sample size was 458 (344 non-disabled students and 114 mentally Disabled students) were enrolled in the study using a simple random sampling technique.

First, the total number of disabled and non-disabled students were obtained from each selected school. Second, the required sample sizes of both mentally disabled and non-disabled students were distributed independently among the selected schools proportionally based on the total number of students in each school. Then, the number of students required to be enrolled was allocated proportionally based on grade level.

Finally, study participants were selected by simple random sampling technique (Random Number Generator) using a list of the students as a sampling frame. Students’ were identified, and their parents contacted by teachers or children. Accordingly, 44, 11, 43 and 16 disabled students and 146, 52, 12 and 134 non-disabled students were sampled from Shibit, Yekatit 23, Felege Abay and Teyma primary schools.

### Operational definitions

**Ova or Parasite seen/positive/:** One of the parasites, eggs, cyst, trophozoite, larva or more than one of this diagnostic stage of intestinal parasites was found in study participants stool sample.

**Water contact:** a student who has playing, swimming, fishing and/or irrigation experience on the various source of water (like lakes, rivers, ponds).

**Mentally Disabled:** students registered as having an intellectual problem in the selected schools or students who attained their education as special needs due to mental impairment will be considered as mentally Disabled students.

**Pit latrine** is a type of toilet that collects human feces in a hole in the ground.

**Flush toilet** is a toilet that disposes of human excreta (urine and feces) by using water to flush it through a drainpipe to another location for disposal, thus maintaining a separation between humans and their excreta.

**A Pour-flush toilet** is like a regular cistern flush toilet except that the water is poured in by the user, instead of coming from the cistern above.

### Data collection tools and procedures

The data for this study were collected from November 1 to November 30, 2018, using both a structured questionnaire adopted from different kinds of literature and for laboratory data with direct stool microscopy. Four data collectors (2 clinical Nurses for the collection of data related to the associated factor and 2 laboratory technicians for direct stool microscopy) and two supervisors (one health officer and one laboratory technologist) were assigned. One day prior to our cross-sectional parasitological and questionnaire surveys a written informed consent form for the parent/guardian of participating children were left with the teachers and distributed to eligible students’ parents/guardians through students or through teachers. During the school-based survey, the signed informed consent sheets were cheeked, unique identification numbers assigned to each participating student and the same number of plastic bottles (stool cup) given for stool collection. A short interview held with each student/parent/guardian, using a questionnaire pertaining to hygiene behavior, drinking water and sanitation. After completing the interview, the participants were asked to give one thumb size of a stool sample and orientation given how to collect the stool sample for each participant, and stool samples were collected.

Laboratory technicians did a stool microscopic examination soon (within 30 min) not to miss the trophozoite of protozoa by using a direct wet mount [17]. The rest samples were preserved with 10% formalin to process using the formol-ether concentration technique [17].

In Bahir Dar Model Sexual and Reproductive Health Clinic Laboratory, 10% formalin, Ether, distilled water/normal saline, cone-shaped test tubes, Microscope, and centrifuge were used to process stool samples. Finally, left-over samples were decontaminated with appropriate bleach and discarded to Bahir Dar Model Sexual and Reproductive Health Clinic incinerator.

### Data management/processing and analysis procedures

Data were coded, entered and cleaned, using EpiData version 3.1 and exported to Statistical package for social science (SPSS) version 23 for analysis. Frequencies and percentages were generated. Tables and graphs used for data presentation. The bi-variable logistic regression method was used to select candidate variables. Independent variables resulting in a *p*-value of less than 0.2 on bi-variable analysis were considered in the multivariable logistic regression analysis for further analysis. Multivariable logistic regression with the forward method was carried out to identify factors associated with IPIs. Adjusted odds ratio (AOR) with 95% Confidence interval (CI) at 5% level of significance was used to measure the strength and significance of the association. Hosmer and Lemeshow goodness of fit test was used for checking the logistic regression model fitting assumption, the assumption was fulfilled at *x*^*2*^ = 7.809 and *P* value = 0.452 since it was > 0.05.

## Results

### Socio-demographic characteristics of the respondents

A total of 418, 104 mentally disabled and 314 non-disabled primary school students participated in the study with 91.23 and 91.28% response rate for disabled and non-disabled students respectively. Of which females account 41.30% (*n* = 43) from mentally disabled students and 55.10% (*n* = 173) from non-disabled students. The mean age of study participants was 14.05 ± 3.66 and 11.96 ± 2.94 for mentally disabled students and non-disabled students respectively [Table 1].

**Table 1:** Socio-Demographic Characteristics of mentally disabled and non-disabled primary school students’ and their families in Bahir Dar City, Amhara Regional State, Ethiopia, 2018 (*n* = 418)

Characteristics		Students with disability status					
		Disabled students(*n* = 104)		Non-disabled students(*n* = 314)		Total(*n* = 418)	
Variables	Category	Freq (%)	*X* ^*2*^ *, p*	Freq (%)	*X* ^*2*^ *, p*	Freq (%)	*X* ^*2*^ *, p*
Mental disability	Yes	104(100)	*–*	0	*–*	104(24.9)	*X* ^*2*^ *= 7.730, P = 0.005*
	No	0		314(100)		314(75.1)	
Age	<=9	15(14.4)	*X*^*2*^ *= 9.368*, *p = 0.009*	88(28.0)	*X*^*2*^ *= 12.608*, *P = 0.002*	103(24.6)	*X*^*2*^ *= 11.317*, *P = 0.003*
	10–14	38(36.5)		151(48.1)		189(45.2)	
	> = 15	51(49.0)		75(23.9)		126(30.1)	
Sex	Male	61(58.7)	*X*^*2*^ *= 0.926*, *p = 0.336*	141(44.9)	*X*^*2*^ *= 4.378*, *P = 0.036*	202(48.3)	*X*^*2*^ *= 6.692*, *P = 0.010*
	Female	43(41.3)		173(55.1)		216(51.7)	
Religion	Orthodox	81(77.9)	*X*^*2*^ *= 1.043*, *p = 0.791*	234(74.5)	*X*^*2*^ *= 3.742*, *P = 0.291*	315(75.4)	*X*^*2*^ *= 4.372*, *P = 0.224*
	Catholic	5(4.8)		17(5.4)		22(5.3)	
	Muslim	11(10.6)		48(15.3)		59(14.1)	
	Protestant	7(6.7)		15(4.8)		22(5.3)	
Grade of students	1–4	104(100)	*–*	138(43.9)	*X*^*2*^ *= 8.088*, *P = 0.004*	242(57.9)	*X*^*2*^ *= 14.555*, *P < 0.000*
	5–8	0(0)		176(56.1)		176(42.1)	
Family size	<=3	3(2.9)	*X*^*2*^ *= 4.703*, *p = 0.319*	71(22.6)	*X*^*2*^ *= 32.357*, *P < 0.000*	74(17.7)	*X*^*2*^ *= 32.265*, *P < 0.000*
	4	22(21.2)		82(26.1)		104(24.9)	
	5	28(26.9)		59(18.8)		87(20.8)	
	6	19(18.3)		53(16.9)		72(17.2)	
	> = 7	32(30.8)		49(15.6)		81(19.4)	
Maternal Education	No formal Education	56(53.8)	*X*^*2*^ *= 8.996*, *p = 0.061*	54(17.2)	*X*^*2*^ *= 2.238*, *P = 0.692*	110(26.3)	*X*^*2*^ *= 10.822*, *P = 0.029*
	Grade 1–8	21(20.2)		113(36.0)		134(32.1)	
	Grade 9–12	18(17.3)		71(22.6)		89(21.3)	
	Collage & above	9(8.7)		76(24.2)		85(20.3)	

NB. Freq= Frequency, %= percent

### Student’s hygiene and safety-related factors

Regards to student’s hygiene and safety 55.80% (*n* = 58) of mentally disabled students and 24.20% (*n* = 76) non-disabled students fingernails had dirty materials. Above half of the mentally disabled students, 52.90% (*n* = 55) had washed their hands sometimes before food while 93.30% (*n* = 293) of non-disabled students did wash their hands always before food [Table 2].

**Table 2:** Hygiene and safety-related characteristics of mentally disabled and non-disabled primary school students’ in Bahir Dar City, Amhara Regional State, Ethiopia, 2018 (*n* = 418)

Characteristics		Students with disability status					
		Disabled students (*n* = 104)		Non-Disabled Students(*n* = 314)		Total (n = 418)	
Variables	Category	Freq (%)	*X* ^*2*^ *, p*	Freq (%)	*X* ^*2*^ *, p*	Freq (%)	*X* ^*2*^ *, p*
Hand washing Habit	water & soap	37(35.6)	*X*^*2*^ *= 8.351*, *p = 0.004*	144(45.9)	*X*^*2*^ *= 30.930*, *P < 0.000*	181(43.3)	*X*^*2*^ *= 41.351, P < 0.000*
	water only	67(64.4)		170(54.1)		237(56.7)	
Hand washing before food	Always	49(47.1)	*X*^*2*^ *= 7.265*, *p = 0.007*	293(93.3)	*X*^*2*^ *= 1.187*, *P = 0.276*	342(81.8)	*X*^*2*^ *= 14.269*, *P < 0.000*
	Sometimes	55(52.9)		21(6.7)		76(18.2)	
Hand washing after latrine	Always	40(38.5)	*X*^*2*^ *= 5.365*, *p = 0.068*	113(36.0)	*X*^*2*^ *= 19.956*, *P < 0.000*	153(36.6)	*X*^*2*^ *= 22.789*, *P < 0.000*
	Sometimes	41(39.4)		132(42.0)		173(41.4)	
	Never	23(22.1)		69(22.0)		92(22.0)	
Fingernail cutting	> = 2 times per week	25(24.0)	*X*^*2*^ *= 2981*, *p = 0.225*	102(32.5)	*X*^*2*^ *= 26.686*, *P < 0.000*	127(30.4)	*X*^*2*^ *= 18.719*, *P < 0.000*
	One time per week	46(44.2)		77(24.5)		123(29.4)	
	<=1 time per 2 weeks	33(31.7)		96(30.6)		129(30.9)	
	No need to cut	–		39(12.4)		39(9.3)	
Fingernail cleanliness	Clean	46(44.2)	*X*^*2*^ *= 13.139*, *p < 0.000*	238(75.8)	*X*^*2*^ *= 34.012*, *P < 0.000*	284(67.9)	*X*^*2*^ *= 53.540*, *P < 0.000*
	Not clean	58(55.8)		76(24.2)		134(32.1)	
Shoes wearing at home	Always	32(30.8)	*X*^*2*^ *= 12.541*, *p = 0.002*	97(30.9)	*X*^*2*^ *= 6.362*, *P = 0.042*	129(30.9)	*X*^*2*^ *= 12.685*, *P = 0.002*
	Sometimes	37(35.6)		142(45.2)		179(42.8)	
	Never	35(33.7)		75(23.9)		110(26.3)	
Shoe wearing at school	Always	83(79.8)	*X*^*2*^ *= 4.059*, *p = 0.044*	314(100)	*–*	397(95.0)	*X*^*2*^ *= 8.705*, *P = 0.003*
	Sometimes	21(20.2)		0(0)		21(5.0)	
Shower taking	<=1 time per week	77(74.0)	*X*^*2*^ *= 1.946, p = 0.584*	108(34.4)	*X*^*2*^ *= 10.890*, *P = 0.004*	185(44.3)	*X*^*2*^ *= 17.756*, *P < 0.000*
	2 times per week	13(12.5)		153(48.7)		166(39.7)	
	> = 3 times per week	8(7.7)		53(16.9)		61(14.6)	
	Never	6(5.8)		0(0)		6(1.4)	

NB. Freq = Frequency, % = percent

Concerning the availability of latrine in the home, all of them (mentally disabled and non- disabled) students said that they had a latrine at their compound. According to the information provided, 65.40% (*n* = 68) mentally disabled and 52.50% (*n* = 165) non-disabled primary school students had not undergone health checkups for over a year [Table 3].

**Table 3:** hygiene and safety-related characteristics of mentally disabled and non-disabled primary school students in Bahir Dar City, Amhara Regional State, Ethiopia, 2018 (n = 418)

Characteristics		Students with disability status					
		Disabled Students(n = 104)		Non-disabled students(n = 314)		Total(n = 418)	
Variables	Category	Freq (%)	*X* ^*2*^ *, p*	Freq (%)	*X* ^*2*^ *, p*	Freq (%)	*X* ^*2*^ *, p*
Water contact activity	1 time/week	10(9.6)	*X*^*2*^ *= 2.846*, *p = 0.416*	54(17.2)	*X*^*2*^ *= 7.002*, *P = 0.072*	64(15.3)	*X*^*2*^ *= 7.596*, *P = 0.055*
	2–3 times/week	9(8.7)		42(13.4)		51(12.2)	
	> 3 times/week	2(1.9)		48(15.3)		50(12.0)	
	Never	83(79.8)		170(54.1)		253(60.5)	
Latrine utilization	Always	50(48.1)	*X*^*2*^ *= 0.879*, *p = 0.644*	314(100)	*–*	364(87.1)	*X*^*2*^ *= 6.525*, *P = 0.038*
	Sometimes	41(39.4)		0(0)		41(9.8)	
	Never	13(12.5)		0(0)		13(3.1)	
Type of latrine at home	Dry latrine	35(33.7)	*X*^*2*^ *= 9.044*, *p = 0.011*	30(9.6)	*X*^*2*^ *= 4.118*, *P = 0.128*	65(15.6)	*X*^*2*^ *= 13.618*, *P = 0.001*
	Flush latrine	25(24.0)		108(34.4)		133(31.8)	
	Pour-flush	44(42.3)		176(56.1)		220(52.6)	
Hand washing facility	No	55(52.9)	*X*^*2*^ *= 2.268*, *p = 0.132*	98(31.2)	*X*^*2*^ *= 5.813*, *P = 0.016*	153(36.6)	*X*^*2*^ *= 10.915*, *P = 0.001*
	Yes	49(47.1)		216(68.8)		265(63.4)	
Defecation modality	Using latrine	45(43.3)	*X*^*2*^ *= 6.816*, *p = 0.033*	314(100)	*–*	359(85.9)	*X*^*2*^ *= 14.470*, *P = 0.001*
	Open field	41(39.4)		0(0)		41(9.8)	
	Using popo	18(17.3)		0(0)		18(4.3)	
Previous health checks	No	68(65.4)	*X*^*2*^ *= 12.279*, *p < 000*	165(52.5)	*X*^*2*^ *= 13.871*, *P < 0.000*	233(55.7)	*X*^*2*^ *= 26.910*, *P < 0.000*
	Yes	36(34.6)		149(47.5)		185(44.3)	
When do you have a health checkups	< 1 year	28(26.9)	*X*^*2*^ *= 6.907*, *p = 0.009*	97(30.9)	*X2 = 14.501*, *P = 0.002*	125(29.9)	*X2 = 27.154*, *P < 0.000*
	1 year–2 year	5(4.8)		28(8.9)		33(7.9)	
	> 2 years	3(2.9)		24(7.6)		27(6.5)	
	Never	68(65.4)		165(52.5)		233(55.7)	
Ways of transportation	Car/taxi	26(25.0)	*X*^*2*^ *= 1.499*, *p = 0.473*	73(23.2)	*X*^*2*^ *= 26.124*, *P < 0.000*	99(23.7)	*X*^*2*^ *= 22.265*, *P < 0.000*
	Foot	24(23.1)		85(27.1)		109(26.1)	
	Both	54(51.9)		156(49.7)		210(50.2)	

NB. Freq = Frequency, % = percent

### Food and drink establishments’ related factors

In relation with drinking water supply in the home, all of them (mentally disabled and non-disabled students received from tape water while about 9.60% (*n* = 10) of the mentally disabled and 20.70% (*n* = 65) of non-disabled students’ households cooking and sanitation water source were from both tape water and well water [Table 4].

**Table 4:** Food and drink establishments’ related characteristics of mentally disabled and non-disabled primary school students in Bahir Dar City, Amhara Regional State, Ethiopia, 2018 (*n* = 418)

Characteristics		Students with disability status					
		Disabled students (n = 104)		Non-disabled students(*n* = 314)		Total(n = 418)	
Variables	Category	Freq (%)	*X* ^*2*^ *, p*	Freq (%)	*X* ^*2*^ *, p*	Freq (%)	*X* ^*2*^ *, p*
Eating raw meat	<= 1 time per week	28(26.9)	*X*^*2*^ *= 0.254*, *p = 0.881*	61(19.4)	*X*^*2*^ *= 4.624, P = 0.202*	89(21.3)	*X*^*2*^ *= 3.880, P = 0.275*
	2–3 times per week	12(11.5)		50(15.9)		62(14.8)	
	> 3 times per week	0(0)		15(4.8)		15(3.6)	
	Never	64(61.5)		188(59.9)		252(60.3)	
Eating raw vegetables	<= 1 time per week	34(32.7)	*X*^*2*^ *= 4.922, p = 0.178*	49(15.6)	*X*^*2*^ *= 11.754, P = 0.008*	83(19.9)	*X*^*2*^ *= 4.274, P = 0.233*
	2–3 times per week	9(8.7)		121(38.5)		130(31.1)	
	> 3 times per week	9(8.7)		59(18.8)		68(16.3)	
	Never	52(50.0)		85(27.1)		137(32.8)	
Eating/consuming/street food	<= 1 time per week	61(58.7)	*X*^*2*^ *= 4.690, p = 0.196*	85(27.1)	*X*^*2*^ *= 5.255, P = 0.154*	146(34.9)	*X*^*2*^ *= 4.113, P = 0.250*
	2–3 times per week	16(15.4)		94(29.9)		110(26.3)	
	> 3 times per week	8(7.7)		78(24.8)		86(20.6)	
	Never	19(18.3)		57(18.2)		76(18.2)	
Cooking &sanitation water	Tape water	94(90.4)	*X*^*2*^ *= 0.794, p = 0.373*	249(79.3)	*X*^*2*^ *= 36.351, P < 0.000*	343(82.1)	*X*^*2*^ *= 29.699, P < 0.000*
	Tape & well water	10(9.6)		65(20.7)		75(17.9)	

NB. Freq = Frequency, % = percent

### Prevalence of intestinal parasitic infections

**Fig. 1:**
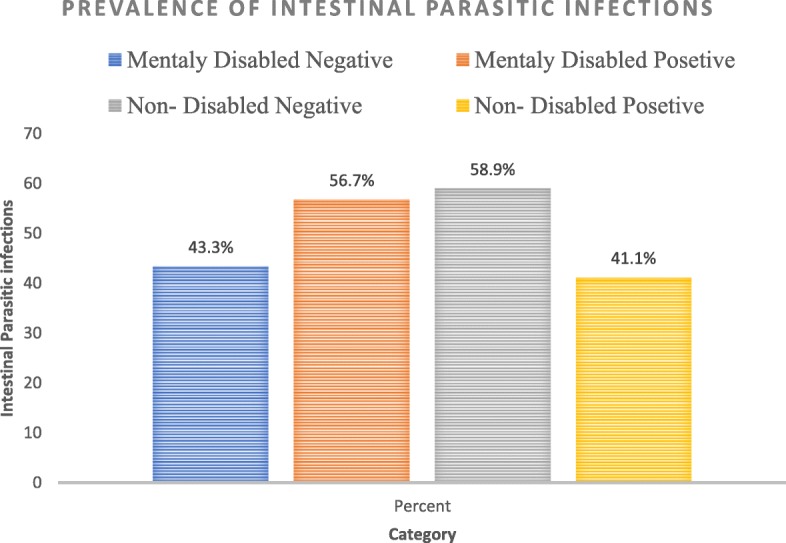
Prevalence of IPIs with respect to disability status in Bahir Dar City, Amhara Regional State, Ethiopia, 2018 (*n* = 418)

Above half of the mentally disabled students, 56.70% (*n* = 59) (95% CI: 47.20–66.20) were infected with at least a single intestinal parasite. While, about 41.10% (*n* = 129) (95% CI: 35.70–46.00) of non-mentally disabled students were infected with intestinal parasites; whereas the overall prevalence of intestinal parasitic infections was 45% (*n* = 188) (95% CI: 40.20–49.80). Point estimate for the difference of the two populations was 15.60% with 95% CI = (0.05, 0.27) [Fig. 1].

### Distribution of IPIs based on mental disability status

Prevalence of single, double and triple parasitic infection was 38.50% (*n* = 161), 25(5.90%) and 2(0.50%), respectively. Ten intestinal parasites were identified from both study groups. Among these single intestinal parasitic infections, *E. histolytica/dispar* was the most prevalent 12.00% (*n* = 50) followed by *A. lumbricoides* 7.90% (*n* = 33), *Hookworm* 6.20% (*n* = 26) and *G. lamblia* 4.80% (*n* = 20) [Table 5].

**Table 5:** Distribution of intestinal parasitic infections among mentally disabled and non-disabled primary school students in Bahir Dar, Amhara Regional State, Ethiopia, 2018

Intestinal parasitic infections		Mentally disabled (*n* = 104)	Non-disabled (*n* = 314)	Total (*n* = 418)
		No. of infections (%)	No. of infections (%)	No. of infections (%)
Helminths	*A. lumbricoides*	10(9.6)	23(7.3)	33(7.9)
	*Hookworm*	7(6.7)	19(6.1)	26(6.2)
	*S. Stercoralis*	4(3.8)	1(0.3)	5(1.2)
	*Taenia species*	1(1.0)	5(1.6)	6(1.4)
	*T. trichiura*	0	3(1.0)	3(0.7)
	*H. nana*	4(3.8)	4(1.3)	8(1.9)
	*E. vermicularis*	4(3.8)	0	4(1.0)
	*S. mansoni*	1(1.0)	5(1.6)	6(1.4)
Protozoa	*E. histolytica*	15(14.4)	35(11.1)	50(12.0)
	*G. lamblia*	6(5.8)	14(4.5)	20(4.8)
Double infections	*A. lumbricoides + E. histolytica*	1(1.0)	10(3.2)	11(2.6)
	*A. lumbricoides + Hookworm*	2(1.9)	3(1.0)	5(1.2)
	*A. lumbricoides +* S. *stercoralis*	1(1.0)	0	1(0.2)
	*Hookworm + E. histolytica*	2(1.9)	5(1.6)	7(1.7)
	*T. trichiura + S. mansoni*	1(1.0)	0	1(0.2)
Triple infections	*A. lumbricoides + Hookworm + E. histolytica*	0	2(0.6)	2(0.5)
Overall infections		59(56.7)	129(41.1)	188(45)

### Factors associated with IPIs among mentally disabled and non-disabled primary school students

In multivariable logistic regression, hand washing habit, fingernail cleanliness, health checkup, family size, the source of cooking and sanitation water, sex of students and grade of students have had a statistically significant association with prevalence of intestinal parasitic infections among primary school students at *P*-value less than 0.05.

Those students who had washed their hands using only water were 2.48 times more likely to develop an intestinal parasitic infection as compared with students who wash his/her hands with water and soap (AOR = 2.48; 1.45,4.12).

Unclean fingernail was predictors’ factors for intestinal parasitic infections for non-disabled students as well as for mentally disabled students. Students who have unclean fingernails were nearly 2.42 times more likely to infected by intestinal parasites than the counterpart (AOR = 2.42; 1.40, 4.17).

Water sources were the strongest determinant factor for IPI on non-disabled students. Students whose household cooking and sanitation water source from both (well and tape) were 4.40 times more likely to develop IPI than students whose source of water from tape water only (AOR = 4.40; 2.32, 8.36).

Those students who had not any medical checkups previously were 1.87 times more likely to be infected with intestinal parasites than mentally disabled students who had medical checkups previously (AOR = 1.87; 95% CI: 1.16,3.02).

The probability of being infected with intestinal parasites for primary school students from a family size of 7 and above was higher than those from a family size of less than or equal to 3 (AOR:2.63; 95%CI:1.209–5.72).

Students in a grade of 1–4 were more likely to be infected with intestinal parasitic infections than those in a grade of 5–8 (AOR: 2.20; 95%CI: 1.37–3.54).

Male primary school students were 1.642 times more likely to be positive for IPIs than females (AOR: 1.64; 95%CI: 1.03–2.63) [Tables 6 and 7**]**.

**Table 6:** Bi-variable and Multivariable analysis of IPI among mentally disabled and non-disabled students in Bahir Dar City, Amhara Regional State, Ethiopia

Variables	Category	IPI		COR (95%CI)	AOR (95%CI)
		Yes, N (%)	No, N (%)		
Mental disability	Non-disabled	129(30.9)	185(44.2)	1	–
	Disabled	59(14.1)	45(10.8)	1.880(1.201, 2.944) **	–
Age	–	188(45)	230(55)	0.919(0.865,0.976) **	–
Sex of students	Male	104(24.9)	98(23.4)	1.668(1.131,2.459) *	1.642(1.026,2.627) *
	Female	84(20.1)	132(31.6)	1	1
Grade of Students	1st cycle [1–4]	128(30.6)	114(27.3)	2.171(1.454,3.242) ***	2.272(1.408,3.666) **
	2^ed^ cycle [5–8]	60(14.4)	116(27.8)	1	1
Family size	<=3	22(5.3)	52(12.4)	1	1
	4	34(8.1)	70(16.7)	1.148(0.602,2.189)	1.015(0.484,2.131)
	5	39(9.3)	48(11.5)	1.920(0.999,3.691)	1.211(0.565,2.595)
	6	38(9.1)	34(8.1)	2.642(1.338,5.215) *	1.747(0.794,3.841)
	> = 7	55(13.2)	26(6.2)	5.000(2.526,9.896) ***	2.736(1.249,5.994) *
Maternal Education	No formal education	61(14.6)	49(11.7)	2.282(1.275, 4.086) **	–
	1–8	64(15.3)	70(16.7)	1.676(0.958, 2.932)	–
	9–12	33(7.9)	56(13.4)	1.080(0.582, 2.006)	–
	College & above	30(7.2)	55(13.2)	1	–
Hand washing Habit	Water & soup	49(11.7)	132(31.6)	1	1
	Water only	139(33.3)	98(23.4)	3.821(2.517,5.801) ***	2.476(1.489,4.119) ***
Hand washing before food	Always	139(33.3)	203(48.6)	1	–
	Sometimes	49(11.7)	27(6.5)	2.650(1.581, 4.444) ***	–
Hand washing habit after latrine	Always	48(11.5)	105(25.1)	1	–
	Sometimes	83(19.9)	90(21.5)	2.017(1.282, 3.175) **	–
	Never	57(13.6)	35(8.4)	3.562(2.072, 6.126) ***	–
Fingernail cutting	> = 2 times per week	39(9.3)	88(21.1)	1	–
	1 time per week	55(13.2)	68(16.3)	1.825(1.087, 3.064) *	–
	1 time per 2 weeks	73(17.5)	56(13.4)	2.941(1.761, 4.914) ***	–
	No need of to cut	21(5.0)	18(4.3)	2.632(1.264, 5.484) *	–
Fingernail cleanliness	Clean	93(22.2)	191(45.7)	1	1
	Not clean	95(22.7)	39(9.3)	5.003(3.198,7.827) ***	2.416(1.400,4.168) **

Note: *statistically significant at *P* < 0.05, ** < 0.01, *** < 0.001

**Table 7:** Bi-variable and Multivariable analysis of IPI among mentally disabled and non-disabled students in Bahir Dar City, Amhara Regional State, Ethiopia

Variables	Category	IPI		COR (95%CI)	AOR (95%CI)
		Yes, N (%)	No, N (%)		
Shoe wearing habit at home	Always	48(11.5)	81(19.4)	1	–
	Sometimes	75(17.9)	104(24.9)	1.217(0.765, 1.936)	–
	Never	65(15.6)	45(10.8)	2.437(1.447, 4.106) **	–
Shoe wearing habit at school	Always	172(41.1)	225(53.8)	1	–
	Sometimes	16(3.8)	5(1.2)	4.186(1.504, 11.651) **	–
Frequency of shower	<= 1 times per week	106(24.5)	85(20.3)	2.060(1.141, 3.721) *	–
	2 times per week	59(14.1)	107(25.6)	0.911(0.496, 1.673)	–
	> = 3 times per week	23(5.5)	38(9.1)	1	–
Water contact activity	1 time per week	28(6.7)	36(8.6)	0.809(0.466,1.405)	–
	2–3 times per week	22(5.3)	29(6.9)	0.789(0.430,1.447)	–
	> 3 times per week	14(3.3)	36(8.6)	0.405(0.208, 0.786) **	–
	Never	124(29.7)	129(30.9)	1	–
Latrine utilization	Always	155(37.1)	209(50)	1	–
	Sometimes	25 (6)	16(3.8)	2.107(1.088, 4.080) *	–
	Never	8(1.9)	5(1.2)	2.157(0.692, 6.722)	–
Type of latrine used at home	Dry/pit latrine	41(9.8)	24(5.7)	2.050(1.160, 3.623) *	–
	Flash latrine	47(11.2)	86(20.6)	0.656(0.421, 1.022)	–
	pour flash latrine	100(23.9)	120(28.7)	1	–
Hand washing facility around the latrine	No	85(20.3)	68(16.3)	1.966(1.313,2.943) **	–
	Yes	103(24.6)	162(38.8)	1	
Ways of defecation	Using latrine	148(35.4)	211(50.5)	1	–
	Open field	28(6.7)	13(3.1)	3.071(1.539, 6.125) **	–
	Using popo	12(2.9)	6(1.4)	2.851(1.047, 7.768) *	–
Health checkup	No	131(31.3)	102(24.4)	2.884(1.923,4.326) ***	1.869(1.156,3.023) *
	Yes	57(13.6)	128(30.6)	1	1
Modes of transportation	Car	32(7.7)	67(16.0)	1	–
	Foot	69(16.5)	40(9.6)	3.612(2.035, 6.410) ***	–
	Both	87(20.8)	123(29.4)	1.481(0.896, 2.448)	–
Source of cooking & sanitation water	Tap water	133(31.8)	210(50.2)	1	1
	well & Tape	55(13.2)	20(4.8)	4.342(2.490,7.571) ***	4.404(2.319,8.364) ***

Note: * statistically significant at *P* < 0.05, ** < 0.01, *** < 0.001

## Discussion

Epidemiological investigations on the prevalence of intestinal parasitic infection and associated risk factors in primary school students are necessary to design appropriate intervention strategies. This study determined the prevalence of intestinal parasitic infections and associated factors among mentally disabled and non-disabled primary school students in Bahir Dar, Amhara Regional State, Ethiopia. The study revealed that 56.70% (95% CI: 47.20–66.20) of mentally disabled and 41.10% (95% CI: 35.70–46.50) non-disabled primary school students were infected with intestinal parasitic infections. Prevalence of intestinal parasitic infections was higher among mentally disabled students than non-disabled students with point estimate for the difference is 15.60% (95% CI: 0.05, 0.27). This suggested that mental disability can directly influence hygiene and safety habits and, consequently, favor the acquisition of intestinal parasitic infections.

The prevalence of IPI among mentally disabled students in this study 56.7% was higher than else studies conducted in Rasht, Northern Iran (5.15%) [18], Brazil (8.30%) [19], Tanzania (12.45%) [20] and Egypt (43.50%) [14]. Because it might be due to poor shoe wearing practices, poor hand washing habits, open defecation practices, poor personal and environmental hygiene and limited health checkup.

In another way, this study was lower than the study conducted in Iran among elderly and mentally retarded residence (78.70%) [21]. This might be due to the fact that the study participants in the latter study is carried out among elderly individuals in the community while the current study done on mentally disabled students at school which indicate most of the mentally disabled students at school may have better follow-up on their personal hygiene and sanitation through teachers and guardian than elderly mentally disabled individuals in the community which directly affect the prevalence of intestinal parasitic infections.

Similarly, the prevalence of IPI among non-disabled students in this study (*P* = 41.10, 95% CI: 35–46.20%) was similar with a previous study conducted in Turkey 44.6% [22], Southern Ethiopia 39.9% [10] and in Amhara Region, Tillili (44.2%) [23]. However, this finding was lower than most studies done worldwide (Peru 47% [24], Argentina 78.30% [25] and Yemen 54.80% [26]) in Africa (South Africa 64.80% [8], Rwanda 50.50% [9], Sao Tome 64.70% [27] and Tanzania 48.70% [28]) and in Ethiopia,(Chencha Town, 81% [11], Jimma, 48.40% [29], Mizan Aman Town, 76.70% [30], and Bahir Dar 65.50% [16]). The most possible reasons were an improvement in proper and protected drinking water supply, better shoe wearing practices at school and improvement on personal and environmental hygiene and sanitation.

In addition to determining the PIPIs, this study also assesses various risk factors of intestinal parasitic infections among mentally disabled and non-disabled students.

The study revealed that unclean fingernails/having dirty materials in fingernails made students be more infected with intestinal parasites (AOR = 2.42;1.40,4.17). A Comparable association of intestinal parasitic infection with the availability of dirty material in fingernail were reported in different studies conducted in different period time and different parts of Ethiopia [16, 31–34]. This could be described by the availability of dirty materials in fingernails may lead to direct feco-oral transmission of intestinal parasite or it uses as habitat to proceed the life cycle of soil-transmitted intestinal parasites.

The previous medical checkup was one of the risk factors for intestinal parasitic infections. Students who had not any medical checkups previously were more likely to be infected with intestinal parasites than students who had medical checkups previously (AOR = 1.87;1.16,3.02), Which is in line with a study conducted in Aksum, Ethiopia [35]. This might indicate mentally disabled students who visit health institution with his/her parents or guardian had a tendency to decrease intestinal parasitic infections through increasing his/her health, safety, and personal hygiene.

The risk of acquiring infections among students who used water only during hand washing was 2.48 times higher than among those who used water and soap during hand washing (AOR = 2.48;1.49,4.12) which is congruent with earlier studies conducted in Ethiopia [36], [29]. This might due to using water only didn’t sufficient to keep our safety and hygiene, so students must use water with soap to remove infective stage of the parasite or dirty materials from those hand.

Students whose household cooking and sanitation water source from both (well and tape) were 4.40 times more likely to develop IPI than students whose source of water from tape water only (AOR = 4.40; 2.32, 8.36), this result also inconsistent with [16, 29, 37]. The possible explanations of the association of water source and increase parasitic infection might be due to an increased chance of contamination of well water by infective stages of different intestinal parasites.

The probability of being infected by intestinal parasites was increased by about 2.74 folds among students belonging in a family size of > = 7 as compared with students belonging in <=3 family size (AOR = 2.74; 1.25, 5.99). Other studies in agreement with this study conducted in Ethiopia were [18] and [33]. This association might be due to the fact that personal hygiene, environmental sanitation, and other nutritional related problems. As a family size increase, there might be a problem of overcrowding, under-nutrition, poor sanitation, and personal hygiene which enhance intestinal parasitic infection susceptibility.

Furthermore, males were 1.64 times high risk for intestinal parasitic infections than females (AOR = 1.64; 1.03, 2.63). This is comparable to a study conducted in Southwest Ethiopia [29]. Male students usually play outdoors and participate in outdoor activities compared to females, which may enhance the risks of IPI. However, this finding contradicts with another study conducted in Ethiopia [30].

Lastly, there was a significant association between the educational level of students and the rate of intestinal parasitic infections. Students in the first cycle were 2.27 times more likely to be infected with intestinal parasitic infections than those in the grade level of the second cycle (AOR = 2.27; 1.41, 3.67). This was in line with other studies [23, 36]. This finding might be due to lack of regular health education program in the school which can decrease their awareness in the prevention and control mechanisms.

## Strength and limitation of the study

### Strength of the study

Both wet mount and formol-ether concentration techniques were used to examine the presence of intestinal parasite from study participants stool sample which used to increase the validity of the measurement of the dependent variable.

The overall ability of direct wet mount microscopy to correctly diagnose intestinal helminths (Test Efficacy = (TP + TN)/ (TN + TP + FN + FP)) was 94%, While, its sensitivity (S = TP/ TP + FN) and negative predictive value (PPV = TP/ (TP/FP) were 76 and 92.7% respectively by taking formol-ether concentration techniques as gold standard method [38].

### Limitation of the study

First, in this study, an interview was held with each student for non-disabled students while parents/guardian/ were asked about their mentally disabled children using a questionnaire pertaining to factors associated with intestinal parasitic infections.

Second, as we only examined a single stool samples by both wet mount and concentrations technique of each study participant, we might underestimate the true prevalence of parasitic infections, due to cyclical nature of life cycle of intestinal parasites, to say negative stool sample at list three consecutive samples in three consecutive days must be examined from each study participants.

## Conclusion

The present study concludes that the prevalence of intestinal parasitic infection among mentally disabled students was higher than the prevalence of intestinal parasitic infections among non-disabled primary school students. However, the prevalence of intestinal parasitic infection among non-disabled students was still high.

Among the different potential risk factors assessed in the study, fingernail cleanliness, previous health checkup, hand washing habits, cooking and sanitation source of water, family size, sex, and educational level were had a statistically significant association with intestinal parasitic infections among primary school students (Additional file 1).

## Additional file

Additional file 1: Laboratory diagnosis of intestinal parasites. (DOCX 18 kb)

## Data Availability

The data can be accessed from the corresponding author through the following address fentahun143@gmail.com // agumlt@yahoo.com. The data will be accessed for research purpose and this is because, during the ethical clearance process, we agree with the Institutional review board of Bahir Dar University to keep the confidentiality of the data set.

## Abbreviations

AOR: Adjusted Odd Ratio
CI: Confidence Interval
IP: Intestinal Parasites
IPI: Intestinal Parasitic Infection
PIPI: Prevalence of Intestinal Parasitic Infection
SPSS: Statistical Package for Social Science
