# Supplementary material for: Intestinal parasitic infections and associated factors among mentally disabled and non-disabled primary school students, Bahir Dar, Amhara regional state, Ethiopia, 2018: a comparative cross-sectional study
Source: BMC Infect Dis. 2019 Jun 21;19:549. doi: 10.1186/s12879-019-4165-2 (PMC6588938; doi:10.1186/s12879-019-4165-2)
Supplement: Supplementary file 1 — Laboratory diagnosis of intestinal parasites. (DOCX 18 kb) [file 12879_2019_4165_MOESM1_ESM.docx]

Laboratory diagnosis of intestinal parasites

- Collection and handling of stool Specimen:

The specimen containers should be leak-proof, clean, dry and free from traces of disinfectant and not contaminated with urine or feces. A large teaspoon amount of feces is adequate or about 10 ml of a fluid specimen. The stool specimen must arrive in the laboratory soon after they are collected. If a delay is necessary, use an appropriate type of preservative.

- Materials and Reagents for stool wet mount and Dobell’ iodine solutions Microscopic examination

1. Microscopic slides 7. wooden applicator sticks
2. Physiological saline (0.85% w/ v) 8. Cover slips
3. Dobell's Iodine solutions 9. Microscope
4. Pasteur pipette 10. The disposable glove as needed
5. Ether 11. Round dish with 10% bleach
6. 10% Formalin 12. stool cup and labeling material
7. Procedures for direct stool microscopy (wet mount):
8. Place a drop of physiological saline (0.85% w/v) in the center of the left half of the slide and place a drop of Dobell’s Iodine solution in the center of the right half of the slide.
9. With an applicator stick, pick up a small portion of the feces (Approximately 2 mg, which is about the size of a match head) and put on the drop of saline. Add a similar portion of the stool sample to the drop of iodine.
10. Mix the feces with the drops to form homogeneous suspensions.
11. Cover each suspension with cover slip by holding the cover slip at an angle of 30 degrees touching the edge of the suspension and gently lowering the cover slip onto the slide so that air bubbles are not introduced.
12. Examine the saline preparations using the 10x objective for trophozoite or cyst of intestinal protozoa and for any ova or larva of helminths.
13. Examine the iodine solution preparation using 40x_objective to identify the cyst stages of protozoa. The iodine will stain the nuclei and the glycogen mass of the cyst([17](#_ENREF_17)).
14. Procedures for Formal-Ether concentration technique
15. Take about 2g or 2ml of stool and mix it in about 10 ml of normal saline solution.
16. Filter through two layers of gauze into a centrifuge test tube.
17. Centrifuge for one minute at medium speed (2000-5000rpm) if the supernatant fluids are very cloudy wash the deposit remix it with 10ml of normal saline.
18. Centrifuge for one minute at medium speed and pour off the supernatant.
19. Add 10ml of formaldehyde solution to the sediments.
20. Stir or mix a supernatant well and left it to stand for five minutes.
21. Add 3ml of ether
22. Stopper the tube, turn it on its side and shake vigorously for 30 seconds/1 minute.
23. Remove the stopper carefully and centrifuge for one minute at low speed (1500rpm)
24. Free the layer of debris by rotating the tip of the wooden applicator stick. Pour off all the supernatant fluid.
25. Mix the remaining fluid well with the deposited by tapping the tube gently.
26. Place 2 drops of the deposit on a slide. Add a drop of iodine solution to the second drop of deposit only.
27. Place cover slips over both drops.
28. Examine microscopically the entire preparation using the 10x objectives for eggs of helmets and 40 x objectives for cysts of protozoa.
29. Identify the stages and species of parasites and report the result.

Note: for formalin preserved specimen follows the same steps but in step one we used distilled water instead of normal saline solution ([17](#_ENREF_17)).
